# YWHAE-NUTM2 oncoprotein regulates proliferation and cyclin D1 via RAF/MAPK and Hippo pathways

**DOI:** 10.1038/s41389-021-00327-w

**Published:** 2021-05-04

**Authors:** Wen-Bin Ou, Meijun Z. Lundberg, Shuihao Zhu, Nacef Bahri, Anastasios Kyriazoglou, Liangliang Xu, Ting Chen, Adrian Mariño-Enriquez, Jonathan A. Fletcher

**Affiliations:** 1 Zhejiang Provincial Key Laboratory of Silkworm Bioreactor and Biomedicine, College of Life and Medicine, Zhejiang Sci-Tech University, 310018 Hangzhou, Zhejiang China; 2 Department of Pathology, Brigham and Women’s Hospital, Harvard Medical School, 20 Shattuck Street, Thorn 528, Boston, MA 02115 USA

**Keywords:** Endometrial cancer, Targeted therapies

## Abstract

Endometrial stromal sarcoma (ESS) is the second most common subtype of uterine mesenchymal cancer, after leiomyosarcoma, and oncogenic fusion proteins are found in many ESS. Our previous studies demonstrated transforming properties and diagnostic relevance of the fusion oncoprotein YWHAE–NUTM2 in high-grade endometrial stromal sarcoma (HG-ESS) and showed that cyclin D1 is a diagnostic biomarker in these HG-ESS. However, YWHAE–NUTM2 mechanisms of oncogenesis and roles in cyclin D1 expression have not been characterized. In the current studies, we show YWHAE-NUTM2 complexes with both BRAF/RAF1 and YAP/TAZ in HG-ESS. These interactions are functionally relevant because *YWHAE-NUTM2* knockdown in HG-ESS and other models inhibits RAF/MEK/MAPK phosphorylation, cyclin D1 expression, and cell proliferation. Further, cyclin D1 knockdown in HG-ESS dephosphorylates RB1 and inhibits proliferation. In keeping with these findings, we show that MEK and CDK4/6 inhibitors have anti-proliferative effects in HG-ESS, and combinations of these inhibitors have synergistic activity. These findings establish that YWHAE-NUTM2 regulates cyclin D1 expression and cell proliferation by dysregulating RAF/MEK/MAPK and Hippo/YAP-TAZ signaling pathways. Recent studies demonstrate Hippo/YAP-TAZ pathway aberrations in many sarcomas, but this is among the first studies to demonstrate a well-defined oncogenic mechanism as the cause of Hippo pathway dysregulation.

## Introduction

Uterine mesenchymal neoplasms afflict women across a wide age range. The biology of uterine neoplasms is heterogeneous, as exemplified by endometrial stromal sarcoma (ESS), which is the second most common subtype of malignant uterine mesenchymal tumor, after leiomyosarcoma^1^. Not only do ESS have varied underlying molecular oncogenic mechanisms, but these varied mechanisms are associated with differences in biologic potential, histologic appearance, and clinical behavior^2^. There are no standardized therapies for any of the molecularly defined subtypes of ESS, underscoring the need for biologic insights defining targetable pathways in these cancers.

Endometrial stromal neoplasms comprise several clinicopathological entities, including endometrial stromal nodules, low-grade ESS (LG-ESS), high-grade ESS (HG-ESS), and undifferentiated uterine sarcoma. Endometrial stromal nodules and LG-ESS often contain oncogenic fusions of *JAZF1* with polycomb genes, including *SUZ12*, *PHF1*, and *EPC1*, of which *JAZF1-SUZ12* fusion is most common^3^. By contrast, oncogenic polycomb gene fusions are uncommon in HG-ESS, which instead often contain *YWHAE-NUTM2* fusions or *BCOR* intragenic mutations^1,4^: these oncogenic somatic mutations are associated with aggressive clinical behavior and poor prognosis^5,6^. We previously identified translocation t(10;17)(q22;p13) as the mechanism of *YWHAE-NUTM2* fusion in HG-ESS and we further showed the t(10;17) resulted in two alternative oncogenic fusions, which had been previously indistinguishable based on conventional chromosomal banding studies^1^. These alternative oncogenic events fuse YWHAE to either of two closely related novel proteins, NUTM2A or NUTM2B, both of which are encoded by genes in 10q22^1^. This is the first example of a recurrent oncogenic rearrangement involving a 14–3–3 protein in cancer and the same fusion was subsequently demonstrated in a subset of pediatric renal sarcomas^7^. Notably, the YWHAE-NUTM2 fusions are diagnostically specific for HG-ESS, among uterine sarcomas^1,8^, and are associated with cyclin D1 upregulation^8,9^. HG-ESS with YWHAE-NUTM2 fusion have strong nuclear cyclin D1 expression, which is found rarely – if at all – in other subtypes of ESS, and is likewise uncommon in other gynecologic sarcomas that can enter the differential diagnosis of HG-ESS, such as leiomyosarcoma^1,9^. We demonstrated that YWHAE-NUTM2 was not found in any of 38 LG-ESS or in 827 uterine and non-uterine mesenchymal tumors, other than HG-ESS^1^. Therefore, both YWHAE-NUTM2 fusion and cyclin D1 expression have proven useful as diagnostic immunomarkers for clinically-aggressive ESS^1,9^. However, the mechanisms by which YWHAE-NUTM2 causes cyclin D1 overexpression and HG-ESS oncogenesis have not been characterized.

The 14–3–3 protein family is encoded by seven distinct genes (*YWHAB, YWHAE, YWHAG, YWHAH, YWHAQ, YWHAS*, and *YWHAZ*) which are expressed ubiquitously in mammalian cells. All 14–3–3 proteins function as homo- or hetero-dimers and bind to motifs that contain phospho-serine or phospho-threonine residues^10^. These 14–3–3 interactions are primarily with signaling proteins that regulate transcription, cell cycle checkpoints, apoptosis and differentiation^11,12^. Both oncogenic and tumor suppressor 14–3–3 functions have been described in gynecologic cancers, but this evidence rests primarily on differences in 14–3–3 expression between the cancer cells and their nonneoplastic counterparts^13^. By contrast, YWHAE-NUTM2 evaluations provide the opportunity to evaluate unquestionable oncogenic 14–3–3 mechanisms in the context of a bona fide structurally-defined mutant 14–3–3 oncoprotein. YWHAE-NUTM2 retains the conserved 14–3–3 protein binding domains encoded by exons 2 and 4 of *YWHAE*, and thereby retains YWHAE dimerization and phosphopeptide binding properties^1^. These YWHAE functions are redirected from their usual predominantly cytoplasmic location to the nucleus, due to influence of a NUTM2 bipartite nuclear localization motif^1^.

In the studies reported herein we provide insight into how YWHAE–NUTM2 leads to cyclin D1 overexpression, thereby driving HG-ESS oncogenesis. In these studies, we evaluate whether YWHAE-NUTM2 induces HG-ESS growth and cyclin D1 expression by interacting with RAF1/BRAF and the Hippo effectors YAP/TAZ. The findings unravel oncogenic mechanisms in HG-ESS and provide rationales for targeting RAF/MEK/MAPK and Hippo-YAP/TAZ signaling pathways as therapeutic approaches in HG-ESS with YWHAE-NUTM2.

## Results

### YWHAE-NUTM2 interacts with RAF1 and BRAF

**Fig. 1:**
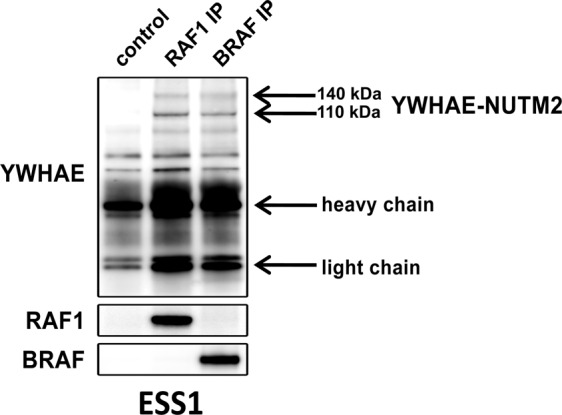
YWHAE-NUTM2 complexes with RAF1 and BRAF in HG-ESS. RAF1 and BRAF immunoprecipitations demonstrate interaction with YWHAE-NUTM2 in ESS1 cells. Normal mouse serum IgG immunoprecipitation is the negative control.

We investigated interaction of YWHAE-NUTM2 with RAF1 and BRAF in ESS1 cells expressing only the endogenous YWHAE-NUTM2 vs. expressing endogenous YWHAE-NUTM2 together with a stably-incorporated lentiviral *YWHAE-NUTM2-FLAG* construct. RAF1 and BRAF were immunoprecipitated from these cells, and the immunoprecipitates were then blotted and immunostained for YWHAE, FLAG, RAF1, and BRAF (Fig. 1 and SFig. 1). These studies demonstrated YWHAE-NUTM2 140/110 kDa isoform complexing with RAF1 and BRAF in ESS1 parental cells and in ESS1 expressing the *YWHAE-NUTM2-FLAG* construct (Fig. 1 and SFig. 1).

### YWHAE-NUTM2 regulates RAF/MEK/MAPK

**Fig. 2:**
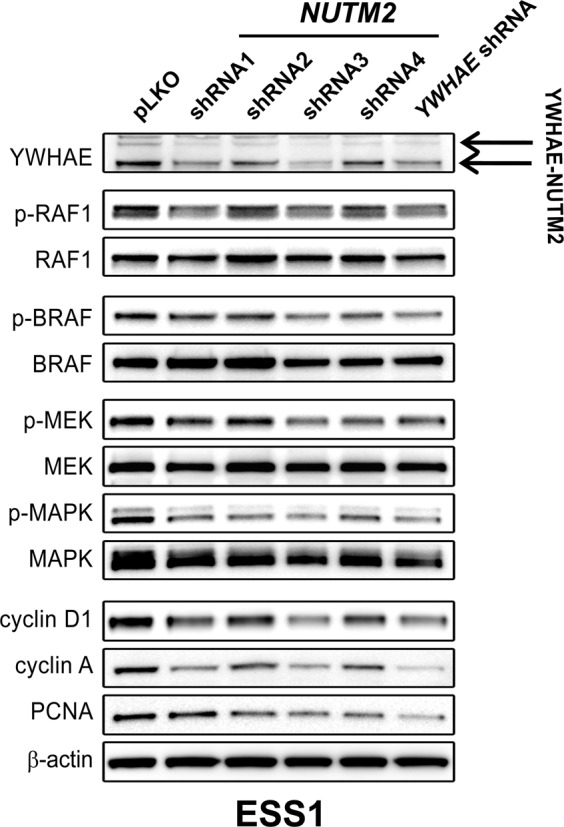
*YWHAE-NUTM2* shRNA knockdown downregulates RAF/MEK/MAPK phosphorylation, cyclin D1, and proliferation markers cyclin A and PCNA. Immunoblotting evaluations were performed in ESS1 cells after 10 days of lentiviral-mediated YWHAE-NUTM2 knockdown and puromycin selection. pLKO is an empty vector control, and the actin stain is a loading control.

**Fig. 3:**
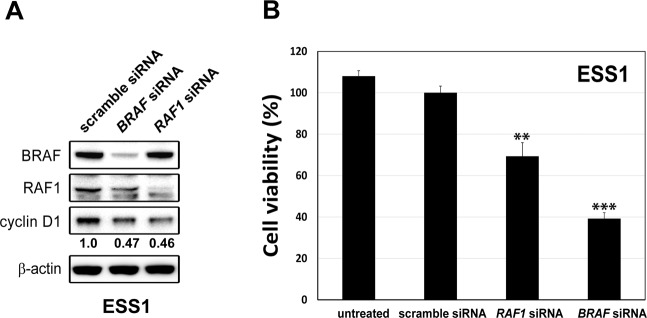
*BRAF* and *RAF1* siRNA knockdowns downregulate cyclin D1 and viability in ESS1 cells. **A** Immunoblotting evaluations were performed in ESS1 cells 4 days after *RAF1* or *BRAF* siRNA transfections (each at 150 nM). The actin stain is a loading control. **B** Cell viability was determined in ESS1 cells 6 days after *RAF1* or *BRAF* siRNA transfections (each at 150 nM) using a Cell-titer Glo® luminescence assay. Data were normalized to the scramble siRNA control and represent the mean values (±s.d.) from quadruplicate cultures, averaged from two independent experiments. Statistically significant differences between scramble siRNA control and *RAF1* siRNA or *BRAF* siRNA treatments are indicated as **p < 0.01, ***p < 0.001.

To address the hypothesis that YWHAE-NUTM2 regulates the RAF/MEK/MAPK pathway, we stably silenced *YWHAE-NUTM2* in ESS1 using lentiviral shRNA constructs. Immunoblotting studies 10 days after the *YWHAE-NUTM2* shRNA transductions and puromycin selection showed greater than 60% inhibition of YWHAE-NUTM2 expression (Fig. 2). This was accompanied by dephosphorylation of RAF1, BRAF, MEK, and MAPK, and inhibition of cyclin D1, cyclin A, and PCNA proliferation expression (Fig. 2). Further studies suggested that cyclin D1 overexpression in YWHAE-NUTM2 ESS is mediated, at least in part, by RAF1 and BRAF. Expression of these RAF kinases was inhibited (>70%) by siRNAs resulted in downregulation of cyclin D1 expression (Fig. 3A). The RAF1 and BRAF siRNA-mediated knockdowns resulted, respectively, in 25 and 60% inhibition of ESS1 viability at 6 days compared with scramble siRNA controls (Promega CellTiter-Glo assay; Madison, WI, USA; Fig. 3B).

### YWHAE-NUTM2 regulates the Hippo pathway

**Fig. 4:**
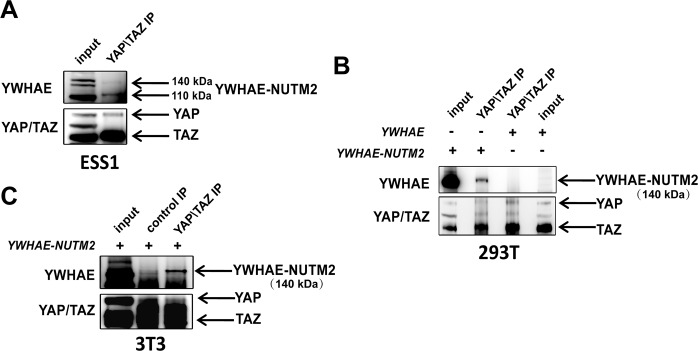
YWHAE-NUTM2 interactions with Hippo pathway effectors YAP and TAZ. **A** YAP/TAZ immunoprecipitation in ESS1 cells immunoblotted for YWHAE. **B** YAP/TAZ immunoprecipitations in 293T cells, 2 days after transfections with *YWHAE-NUTM2* vs. *YWHAE* constructs, immunoblotted for YWHAE. **C** YAP/TAZ immunoprecipitations in 3T3 cells, 2 days after transfection with *YWHAE-NUTM2*. Normal rabbit serum IgG immunoprecipitation (middle lane) is a negative control.

Based on precedent for YWHAE interactions with YAP and TAZ^14–17^, we asked whether YWHAE-NUTM2 interacts with YAP and TAZ in ESS1 cells expressing endogenous YWHAE-NUTM2, and in HEK293T and 3T3 cells 48 h post-transfection with *YWHAE-NUTM2-FLAG* or *YWHAE-HA* constructs. Immunoblotting of YAP and TAZ immunoprecipitations demonstrated YAP/TAZ interactions with YWHAE-NUTM2 (140 kDa band) in ESS1 cells and in the HEK293T and 3T3 cells (Fig. 4A–C and SFig. 2). Notably, YAP/TAZ interactions were not demonstrable with a comparator YWHAE-HA construct in HEK293T cells, suggesting that YAP/TAZ interactions with YWHAE-NUTM2 are stronger than those reported previously for YWHAE (Fig. 4B and SFig. 2).

### YWHAE-NUTM2 regulates cell viability via the Hippo pathway

**Fig. 5:**
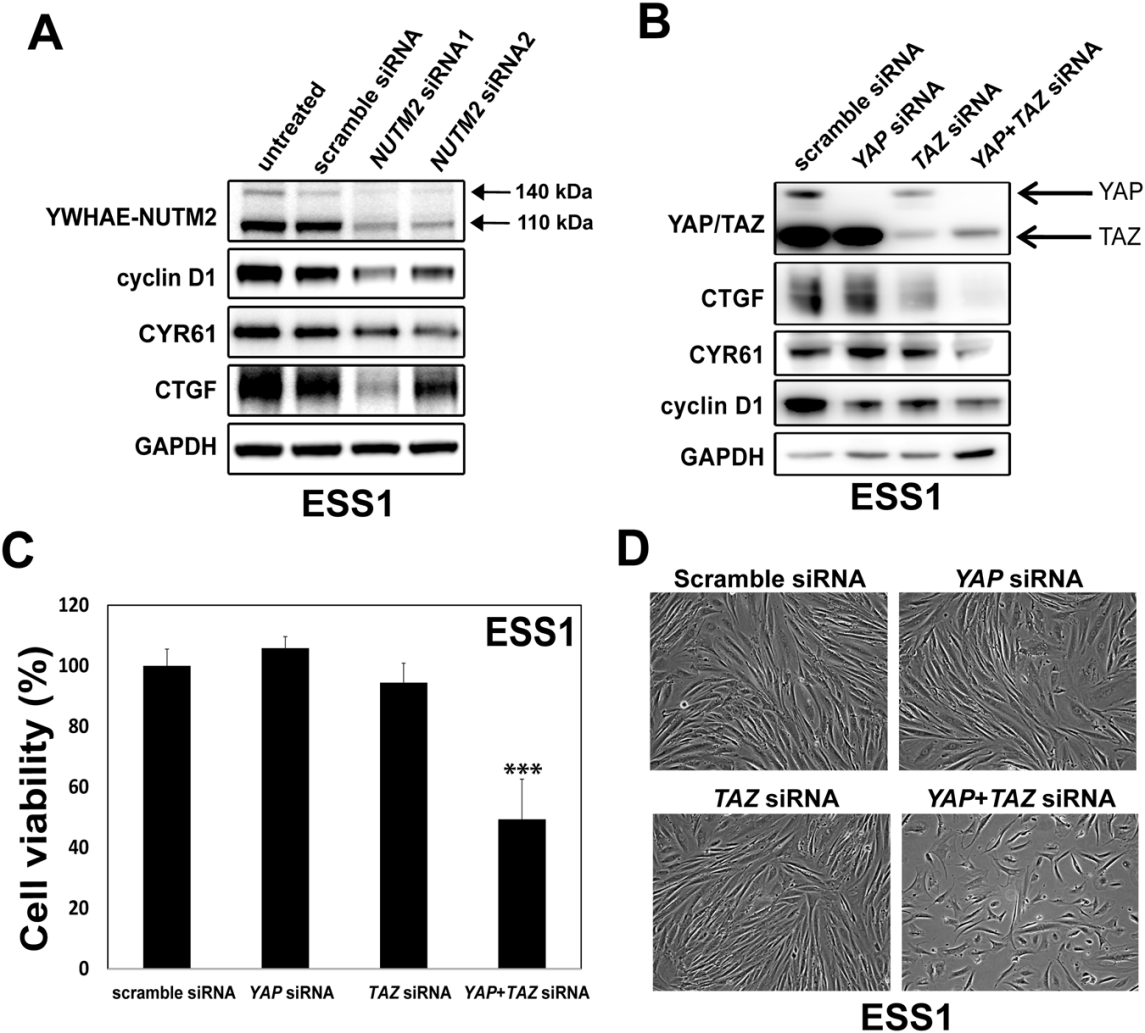
YWHAE-NUTM2 and Hippo pathway functional relationships. **A** Immunoblotting for Hippo effectors was performed in ESS1 cells 5 days after *NUTM2* siRNA transfections (100 nM). The GAPDH stain is a loading control. **B** Immunoblotting was performed in ESS1 cells 5 days after *YAP*, *TAZ*, or *YAP* + *TAZ* siRNA transfections (each at 100 nM). The GAPDH stain is a loading control. **C** Cell viability was determined in ESS1 cells 6 days after *YAP*, *TAZ*, or *YAP* + *TAZ* siRNA transfections (each at 100 nM) using a Cell-titer Glo® luminescence assay. Data were normalized to scramble siRNA control and represent the mean values (±s.d.) from quadruplicate cultures and were averaged from two independent experiments. Statistically significant differences between scramble siRNA control and *YAP*, *TAZ*, or *YAP* + *TAZ* siRNA treatments are indicated as ***p < 0.001. **D** ESS1 growth response 5 days after *YAP*, *TAZ*, or *YAP* + *TAZ* siRNA transfections.

Among uterine sarcomas, cyclin D1 is strongly and uniquely overexpressed in YWHAE-NUTM2 HG-ESS^9^. Cyclin D1 is a key effector of the Hippo pathway. To evaluate whether YWHAE-NUTM2 regulates HG-ESS viability via Hippo/cyclin D1, we silenced *YWHAE-NUTM2*, *YAP*, and *TAZ* in ESS1 with siRNAs, and then determined expression of the Hippo effectors – CTGF, CYR61, and cyclin D1 – by immunoblotting (Fig. 5A, B). siRNA transfections accomplishing >80% downregulation of YWHAE-NUTM2, YAP, or TAZ expression suppressed expression of CTGF, CYR61, and cyclin D1 (Fig. 5A, B). Concomitant siRNA inhibition of both YAP and TAZ inhibited ESS1 cell viability more than inhibition of either gene individually (Fig. 5C and SFig. 3). Similarly, ESS1 six-well monolayer growth was inhibited markedly after concomitant *YAP-TAZ* siRNA knockdown (Fig. 5D). These studies were corroborated by three independent siRNA transfections.

### Cyclin D1 regulates proliferation in ESS

**Fig. 6:**
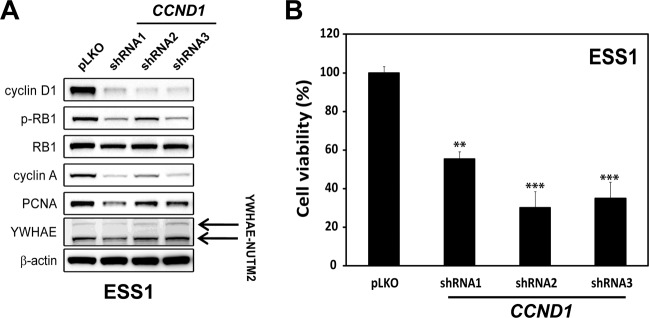
Cyclin D1 regulation of HG-ESS proliferation. **A** Immunoblotting for RB1 dephosphorylation (RB1 activation) and proliferation markers cyclin A and PCNA were performed in ESS1 cells 4 days after *CCND1* shRNA lentiviral knockdown. *YAP*, *TAZ*, or *YAP* + *TAZ* siRNA transfections (each at 100 nM). pLKO is an empty vector control, and the actin stain is a loading control. **B** Cell viability was determined in ESS1 cells 6 days after *CCND1* shRNA lentiviral knockdowns with three independent shRNA sequences. The data were normalized to empty lentivirus (pLKO) control infections and represent the mean values (±s.d.) from quadruplicate cultures, and were averaged from two independent experiments. Statistically significant differences between empty vector control and *CCND1* shRNA are indicated as **p < 0.01, ***p < 0.001.

Because cyclin D1 has varied biological roles, we determined whether *CCND1* manipulations impacted cell growth and biomarkers of ESS cell proliferation. *CCND1* gene expression was silenced by lentiviral shRNA transduction in ESS1 cells, resulting in >90% inhibition of cyclin D1 expression, assessed at 96 h after transduction (Fig. 6A). This inhibition of cyclin D1 expression resulted in inhibition of RB1 phosphorylation, inhibition of proliferation markers cyclin A and PCNA (Fig. 6A), and inhibition of cell viability (Fig. 6B) and wound healing (SFigure 4). However, inhibition of cyclin D1 expression had minimal or no impact on YWAHE-NUTM2 fusion oncoprotein expression. These studies were corroborated by three independent shRNA transductions.

### Synergistic anti-proliferative effects via dual targeting of MEK and CDK4/6

**Fig. 7:**
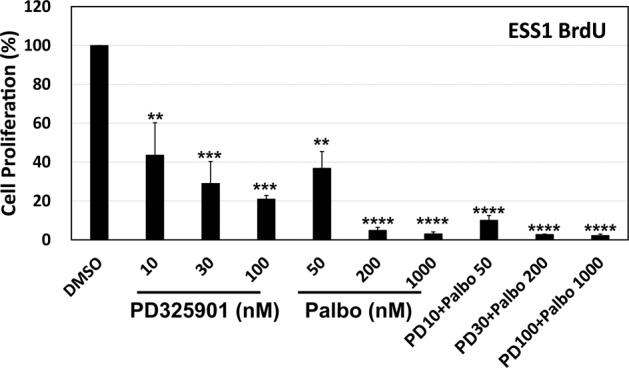
MEK-inhibitor and CDK4/6-inhibitor individual and combined effects on HG-ESS proliferation. ESS1 cells were treated with PD325901, palbociclib, or combined PD325901 and palbociclib at indicated concentrations for 3 days, and labeled with anti-BrdU-POD for the last 24 h. The data were normalized to a DMSO control and represent the mean values (±s.d.) from quadruplicate cultures, and were averaged from two independent experiments. Statistically significant differences between DMSO control and inhibitor treatments are indicated as **p < 0.01, ***p < 0.001, ****p < 0.0001.

Synergistic antagonism of cell proliferation was demonstrated after combined inhibition of MEK and CDK4/6-cyclin D1 in ESS1 cells (Fig. 7). MEK inhibition by PD325901 (10 nM) and CDK4/6 inhibition by palbociclib (50 nM) resulted, respectively, in 55 and 65% reductions in ESS viability, compared to the DMSO control. Dose-response anti-proliferative effects were also demonstrated for both PD325901 and palbociclib. Combination treatment with PD325901 (10 nM) and palbociclib (50 nM) resulted in 90% reduction in ESS1 viability (Fig. 7), **p < 0.01 for 10 nM PD325901 and 50 nM palbociclib treatment alone, and ****p < 0.0001 for combination treatment.

## Discussion

These novel studies demonstrate that cyclin D1 overexpression in HG-ESS depends on YWHAE–NUTM2 activation of RAF/MAPK and Hippo pathways. The rationales for studying RAF/MAPK pathway roles were: (1) MAPK is a known positive regulator of cyclin D1 expression^18^; (2) BRAF and RAF1 heterodimerization is generally required for MEK/MAPK phosphorylation and cell proliferation^19,20^; (3) BRAF and RAF1 complex with YWHAE and other 14–3–3 proteins^19,21,22^;, and (4) precedent for RAF1 translocation from cytoplasm to the nucleus^23–25^ raised the possibility that RAF1 might interact with the predominantly nuclear YWHAE-NUTM2 oncoproteins. The rationales for studying Hippo pathway roles were: (1) upregulation of cyclin D1, along with CTGF and CYR61, is a well-known Hippo function, resulting from transcriptional upregulation by the Hippo effectors YAP and TAZ; (2) YAP and TAZ interact with YWHAE and other 14–3–3 proteins^14–17^;, and (3) our recent studies show that Hippo YAP/TAZ mechanisms induce dramatic cyclin D1 overexpression and cell proliferation in another subtype of mesenchymal neoplasia, gastrointestinal stromal tumor^26^.

The studies reported herein indeed demonstrate YWHAE-NUTM2 interaction with BRAF and RAF1, and with YAP and TAZ. These interactions were demonstrated in a variety of models, including ESS1, which is the only known cell line derived from a YWHAE-NUTM2 HG-ESS (Fig. 1, SFigs. 1 and 2, and Fig. 4). Furthermore, *YWHAE-NUTM2* knockdown in ESS1 inhibited phosphorylation of BRAF, RAF1, MEK, and MAPK, and inhibited expression of cyclin D1 and the proliferation markers cyclin A and PCNA (Fig. 2). Evidence for RAF-MEK pathway roles in HG-ESS oncogenesis included ESS1 dependence on BRAF and RAF1 for viability and cyclin D1 expression (Fig. 3A, B), and on MEK/MAPK for proliferation (Fig. 7).

The above-mentioned findings are consistent with YWHAE-NUTM2 oncogenic roles in RAF/MEK/MAPK pathway dysregulation which then induce ESS proliferation and cyclin D1 overexpression. However, our studies show that HG-ESS cyclin D1 overexpression and cell proliferation do not depend only on RAF1 and BRAF dysregulation, but also result from Hippo dysregulation. The evidence for Hippo oncogenic dysregulation as a mechanism for cyclin D1 upregulation includes inhibition of cyclin D1 and the Hippo signaling effectors CYR61 and CTGF (Fig. 5A) after *YWHAE-NUTM2* silencing, together with the above-mentioned YWHAE-NUTM2 interaction with YAP/TAZ. In keeping with this evidence, YAP and TAZ knockdowns reduced ESS1 viability and inhibited expression of cyclin D1, CYR61, and CTGF (Fig. 5B–D and SFig. 3). Interestingly, YAP and TAZ are transcription factors whose nuclear localization results from novel nuclear localization signals but also from Hippo inactivation, which causes YAP/TAZ-dephosphorylation and release from cytoplasmic retention factors, particularly 14–3–3 proteins, including YWHAE^16^.

Cyclin D1 has varied tumorigenic roles in human cancers which include CDK4/6 complexing to regulate the cell cycle^27^ and RAD51-coregulation of DNA damage repair^28^. These putative oncogenic roles generally result from cyclin D1 overexpression, and the mechanisms of overexpression are largely unknown, although genomic *CCND1* amplification or translocation accounts for overexpression in subsets of human cancer, including breast cancers and lympoma^29,30^. In the studies herein *CCND1* knockdown in ESS1 substantially inhibited cell proliferation, inhibited the cyclin A and PCNA proliferation markers, and inhibited RB1 hyperphosphorylation – indicating restoration of RB1 tumor suppressor function (Fig. 6A, B). Likewise, CDK4/6 inhibition by palbociclib treatment had substantial anti-proliferative effects in ESS1 (Fig. 7). Notably, additive ESS1 anti-proliferative effects resulted from combination treatment with the MEK and CDK4/6 inhibitors (Fig. 7), providing further evidence that both Hippo/cyclin D1 and RAF-MEK pathways are required for YWHAE-NUTM2 oncogenesis.

**Fig. 8:**
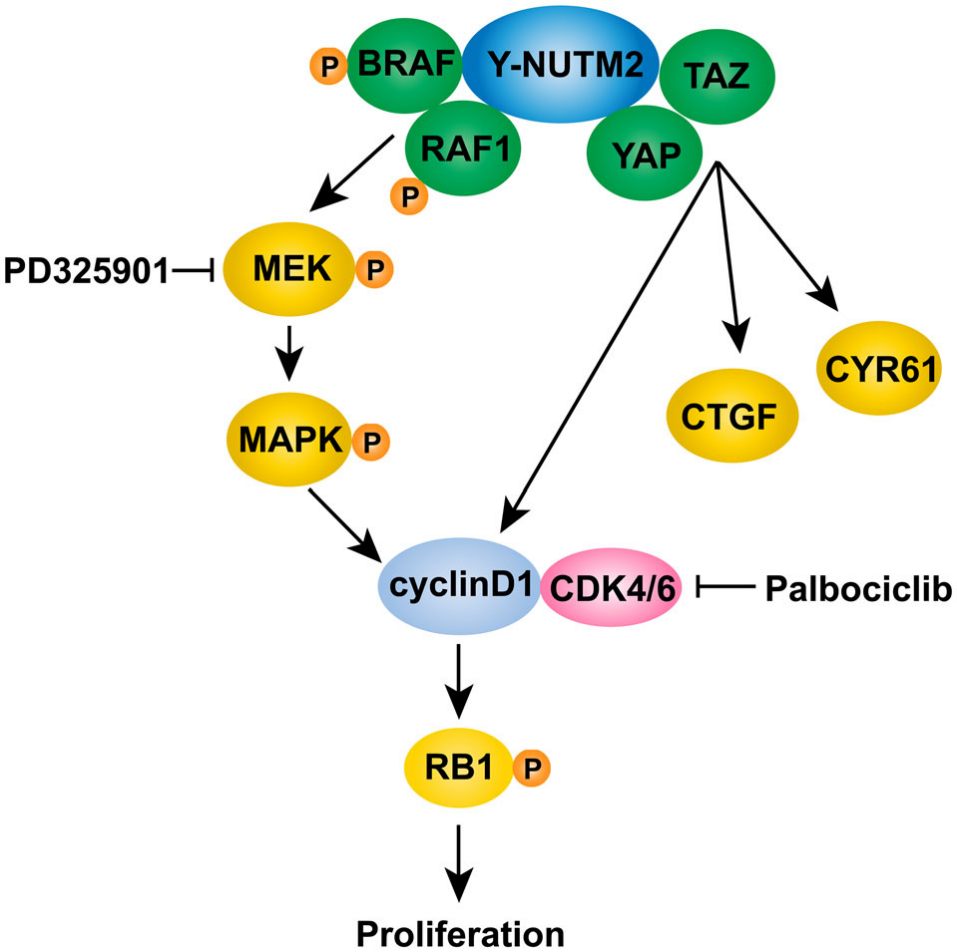
YWHAE-NUTM2 interacts with BRAF/RAF1 and YAP/TAZ in high-grade ESS. YWHAE-NUTM2 oncogenic activation of these pathways regulates cyclin D1 expression and thereby cell proliferation.

In sum, these studies provide the first insights into YWHAE-NUTM2 oncogenic mechanisms and intriguingly show that YWHAE-NUTM2 dysregulates both RAF1/BRAF and the Hippo pathway to cause overexpression of cyclin D1 (Fig. 8), which is a key diagnostic biomarker in HG-ESS. These advances also identify the RAF/MEK/MAPK and Hippo pathways, and CDK4/6, as rational targets for evaluation of therapeutic strategies in HG-ESS with YWHAE-NUTM2 fusions. Although there are no approved targeted therapies for HG-ESS, a recent study demonstrated substantial clinical response to the kinase-inhibitor pazopanib in an ESS patient with *YWHAE-NUTM2* rearrangement^31^. The advances reported herein identify new rationales for targeted therapies in these clinically challenging cancers.

## Materials and methods

### Antibodies and reagents

Polyclonal antibody to YWHAE (HPA008445) was from Sigma-Aldrich (St, Louis, MO). Polyclonal antibodies to p-RB1 (S807/811, #9308), p-RAF1 (S259, #9421), p-BRAF (S445, #2696), p-MEK (S217/221, #9121), p-MAPK (T202/Y204, #9101), MEK (#9122), MAPK (#9102), and YAP/TAZ (#8418) were from Cell Signaling Technology (Beverly, MA), whereas those for RAF1 (sc-7267), CTGF (sc-14939) and CYR61 (sc-13100) were from Santa Cruz Biotechnology (Santa Cruz, CA). Monoclonal mouse antibodies for BRAF (sc-5284), cyclin D1 (sc-20044), and PCNA (sc-56) were from Santa Cruz Biotechnology, for RB1 (#9309) was from Cell Signaling Technology, for cyclin A (NCL-CYCLIN A) was from Novocastra/Leica (Newcastle upon Tyne, UK), and for FLAG (F-1804), GAPDH (G8795), and actin (A4700) were from Sigma-Aldrich. Platinum PCR SuperMix was from Invitrogen Life Technologies (Carlsbad, CA). PD325901 and palbociclib were from LC Laboratories (Woburn, MA).

### Cell culture

The ESS1 cell line was established from a HG-ESS with YWHAE-NUTM2^1^. Cells were screened for mycoplasma using a Mycoplasma Stain Assay Kit (Sigma-Aldrich), and authenticated by SNP array analysis prior to these studies and by RT-PCR and FISH, before and after the studies, to validate presence of the *YWHAE-NUTM2* rearrangement. The ESS1 cell line was maintained in IMDM medium with 15% fetal bovine serum (FBS) containing penicillin/streptomycin and l-glutamine. The studies were conducted in accordance with recognized ethical guidelines (U.S. Common Rule) and were approved by Brigham and Women’s Hospital and Zhejiang Sci-Tech University Institutional Review Boards.

### Fusion construct and cloning

A *YWHAE–NUTM2–FLAG* fusion cDNA containing *Bam*HI (*YWHAE* sequence) and *Eco*RI (*FLAG* sequence) restriction sites was synthesized (GenScript) per the *YWHAE–NUTM2* fusion transcript sequence in ESS1 and cloned in a pUC57 vector. The fusion gene sequence was validated by sequencing. It was further subcloned in pCDNA3(+) by *Eco*RI and *Bam*HI (GenScript). *YWHAE–NUTM2–FLAG* was then subcloned into lentiviral vector pCDH-CMV-MCS-EF1-Puro (System Biosciences) by *Nhe*1 & *Not*1 (New England Biolabs). Construct integrity was verified by Sanger sequencing.

### Transfection

The fusion construct was expressed in 293T or 3T3 cells by Lipofectamine-based transfection according to the manufacturer’s instructions (Invitrogen Life Technologies). Briefly, scrambled control, *NUTM2* siRNAs (s198355 and s195919; Invitrogen Life Technologies), *BRAF* siRNA (5′-AAGUGGCAUGGUGAUGUGGCA-3′, Invitrogen Life Technologies), *RAF1* siRNA (5′-AAUAGUUCAGCAGUUUGGCUA-3′, Invitrogen Life Technologies), *YAP* siRNA (sc-38637), *TAZ* siRNA (sc-38568, Santa Cruz Biotechnology), *YWHAE-NUTM2-FLAG*, or *YWHAE-HA* constructs were incubated with PLUS in serum-free medium for 15 min at room temperature, then mixed in diluted Lipofectamine in equal volumes with scrambled control, siRNAs or construct–PLUS mixtures and incubated for another 15 min at room temperature. Finally, siRNA/construct–PLUS–Lipofectamine complexes were added to 60% confluent ESS1 cells, 293T, or 3T3 cell lines under serum-free medium conditions in 6- or 96-well plates. DNA–PLUS–Lipofectamine complexes in serum-free medium were completely replaced with serum-containing regular medium after a 3-h incubation. Cells were lysed for immunoprecipitation at 48 h, western blot analysis at 96 h, or cell viability assay at 6 days post-transfection. Cell culture images were obtained by using a Spot RT Slider Camera and Spot software (Version 4.6 for Windows) and a Nikon Eclipse TE2000-S inverted microscope. Experiments were performed in triplicate.

### Preparation of lentiviral *CCND1*, *NUTM2*, and *YWHAE* shRNA constructs and lentiviral infections

Lentiviral *CCND1* shRNA constructs (shRNA1, TRCN0000040038; shRNA2, TRCN0000040039; and shRNA3, TRCN0000040042) were from Sigma. *NUTM2* and *YWHAE* shRNAs were from Broad Institute RNAi Consortium: *NUTM2* shRNA1, 5′-TGCTCCTGTGGTGCCTGTTAT-3′; and *NUTM2* shRNA2, 5′-GTGAGTCAGAAGGACAATTTA-3′, *NUTM2* shRNA3, 5′-TCTTGCTGGGCCTTAGCTTTG-3′; and *NUTM2* shRNA4, 5′-TATGTTCCAGGAACCTGTTTA-3′. *YWHAE* shRNA: 5′-CCACAGGTATCTGGCAGAATT-3′. Lentiviral preparations were produced by cotransfecting empty vector pLKO.1 puro with *CCND1*, *NUTM2*, or *YWHAE* shRNAs and helper virus packaging plasmids pCMVΔR8.91 and vsv-g (at a 10:10:1 ratio) into 293T cells. Transfections were carried out with Lipofectamine and PLUS reagent. Lentiviruses were harvested at 24, 36, 48, and 60 h post-transfection and stored at −80 °C.

ESS1 cells were seeded in six-well plates. Infections were carried out in the presence of 8 μg/mL polybrene. After transduction, ESS1 were selected with 2 μg/mL puromycin for 10 days, then lysed for western blot analysis.

### Immunoblotting

Whole cell lysates were prepared using lysis buffer (1% NP-40, 50 mM Tris-HCl pH 8.0, 100 mM sodium fluoride, 30 mM sodium pyrophosphate, 2 mM sodium molybdate, 5 mM EDTA, and 2 mM sodium orthovanadate) containing protease inhibitors (10 μg/mL aprotinin, 10 μg/mL leupeptin, and 1 mM phenylmethylsulfonyl fluoride). Lysates were cleared by centrifugation at 14,000 rpm for 20 min at 4 °C, and lysate protein concentrations were determined using a Bio-Rad protein assay (Bio-Rad Laboratories Hercules, CA, USA). Electrophoresis and western blotting were performed as described previously^32^. The hybridization signals were detected by chemiluminescence (Immobilon Western, Millipore Corporation, MA) and captured using an Amersham Imager 600 chemiluminescence imaging system (GE Healthcare, MA, USA).

### Immunoprecipitation

One mg of protein lysate (500 μL) was preadsorbed for 30 min using 20 μL of sepharose-protein G or A beads at 4 °C. Then 2 μg of primary antibodies to RAF1, BRAF, or YAP/TAZ were rocked with the lysates for 2 h at 4 °C, whereas normal mouse IgG or rabbit IgG was substituted as a comparator group for immunoprecipitation stringency. Then 20 μL of sepharose-protein G/A beads were added and rocked overnight at 4 °C, then centrifuged at 10,000 rpm for 2 min at 4 °C, after which the sepharose beads were washed three times with 750 μL of IP buffer (lysis buffer without protease inhibitors) for 25 min per wash, and were then washed once with 750 μL 10 mM Tris-Cl buffer (pH 7.6). Loading buffer (20 μL) was added to the beads and boiled for 5 min at 95 °C.

### Cell viability analysis

ESS1 cells were plated at 2500 cells/well in a 96-well flat-bottomed plate (Falcon, Lincoln NJ) and cultured in Iscove’s modified Dulbecco’s Medium for 24 h before treatment with siRNA. Cell viability was determined 6 days post-transfection with the CellTiter-Glo luminescent assay from Promega (Mannheim, Germany). Viability was quantified using a Veritas™ Microplate Luminometer from Turner Biosystems (Sunnyvale, CA). Data were normalized to scramble controls. All assays were performed in quadruplicate wells and in triplicate.

### In vitro wound-healing assays

A slash was created in confluent cell cultures, using the tip of a P-100 pipette, at 4 days after *CCND1* shRNA transduction. The plates were photographed at 0 and 72 h with Spot software (Version 4.6 for Windows) and a Nikon Eclipse TE2000-S inverted microscope. Experiments were performed in triplicate.

### BrdU uptake analysis

Cells were plated in 96-well plates at 5000 cells/well in growth medium and incubated overnight. Cells were treated with PD325901 or palbociclib for 72 hours. BrdU was added to the cells for the last 24 hours. Cell labeling with BrdU, fixation and detection were performed using a BrdU Cell Proliferation Assay kit and anti-BrdU-peroxidase (Roche Applied Science, Branford, CT). BrdU incorporation was presented as % of DMSO control. All assays were performed in triplicate wells and were repeated three times.

### Statistical analysis

Student’s *t* tests were performed on data from cells treated with inhibitors/shRNAs/siRNAs or DMSO/pLKO (control). Statistically significant differences between control and treatment were defined as *p < 0.05, **p < 0.01, ***p < 0.001, and ****p < 0.0001.

## Supplementary information

Supplemental Figures
