# Supplementary material for: YWHAE-NUTM2 oncoprotein regulates proliferation and cyclin D1 via RAF/MAPK and Hippo pathways
Source: Oncogenesis. 2021 May 4;10(5):37. doi: 10.1038/s41389-021-00327-w (PMC8097009; doi:10.1038/s41389-021-00327-w)
Supplement: Supplementary file 1 — Supplemental Figures [file 41389_2021_327_MOESM1_ESM.pdf]

SFigure 1

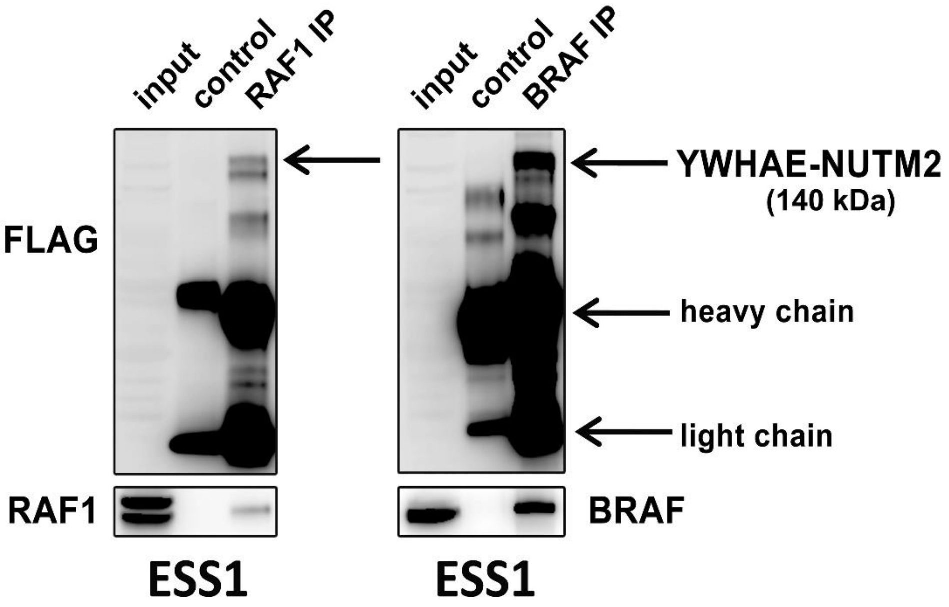

SFigure 1. YWHAE-NUTM2 interactions with RAF1 and BRAF. **A**) Immunoprecipitations of RAF1 (left panel) and BRAF (right panel) from ESS1 cells with lentivirus-mediated *YWHAE-NUTM2-FLAG* stable expression. Normal mouse serum IgG immunoprecipitations (middle lanes) were negative controls.

SFigure 2

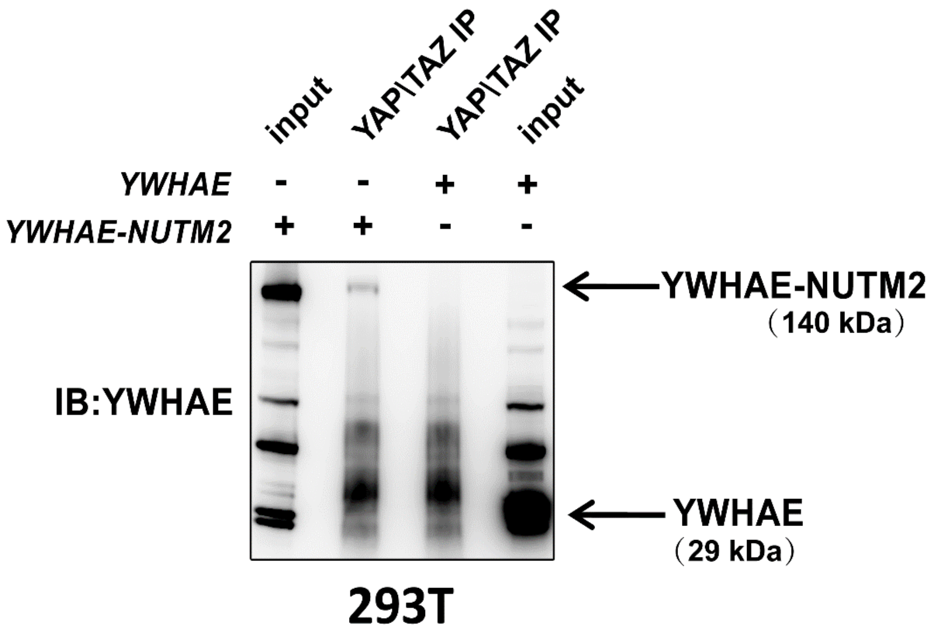

SFigure 2. YWHAE-NUTM2 interactions with Hippo pathway effectors YAP and TAZ. YAP/TAZ immunoprecipitations in 293T cells, 2 days after transfections with *YWHAE-NUTM2* vs. *YWHAE* constructs, immunoblotted for YWHAE.

SFigure 3

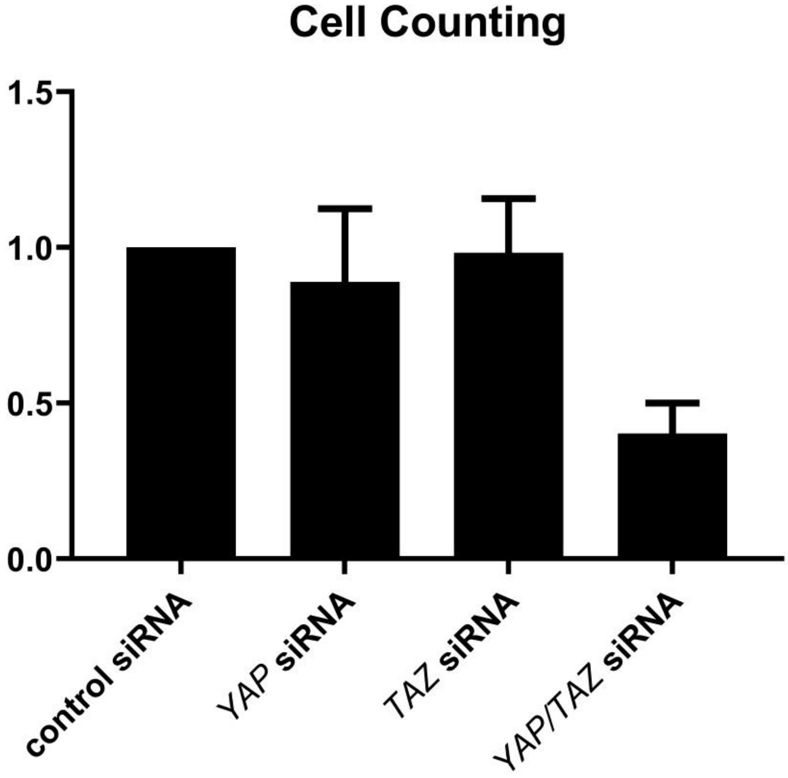

SFigure 3. ESS1 cell counts were determined in ESS1 cells 5 days after transfection with *YAP*, *TAZ*, or *YAP+TAZ* siRNAs (each at 100 nM). Data were normalized to scramble siRNA control and represent the mean values (± s.d.) from quadruplicate cultures and were averaged from two independent experiments.

SFigure 4

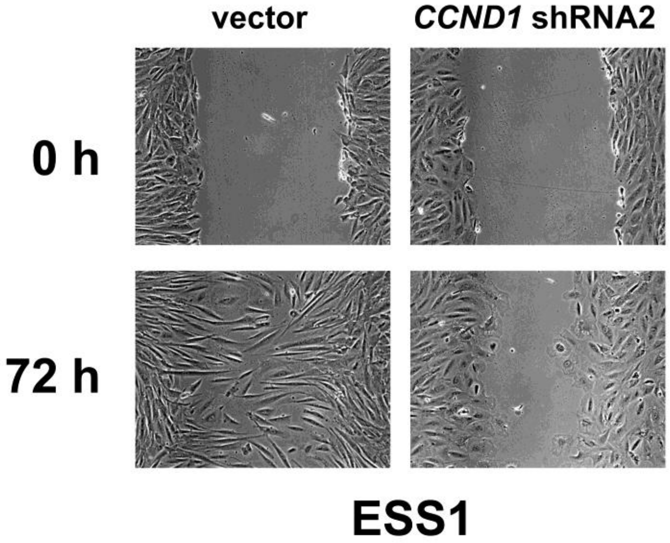

SFigure 4. *In vitro* wound assays demonstrate impact of *CCND1* knockdown on ESS1 cell migration.
